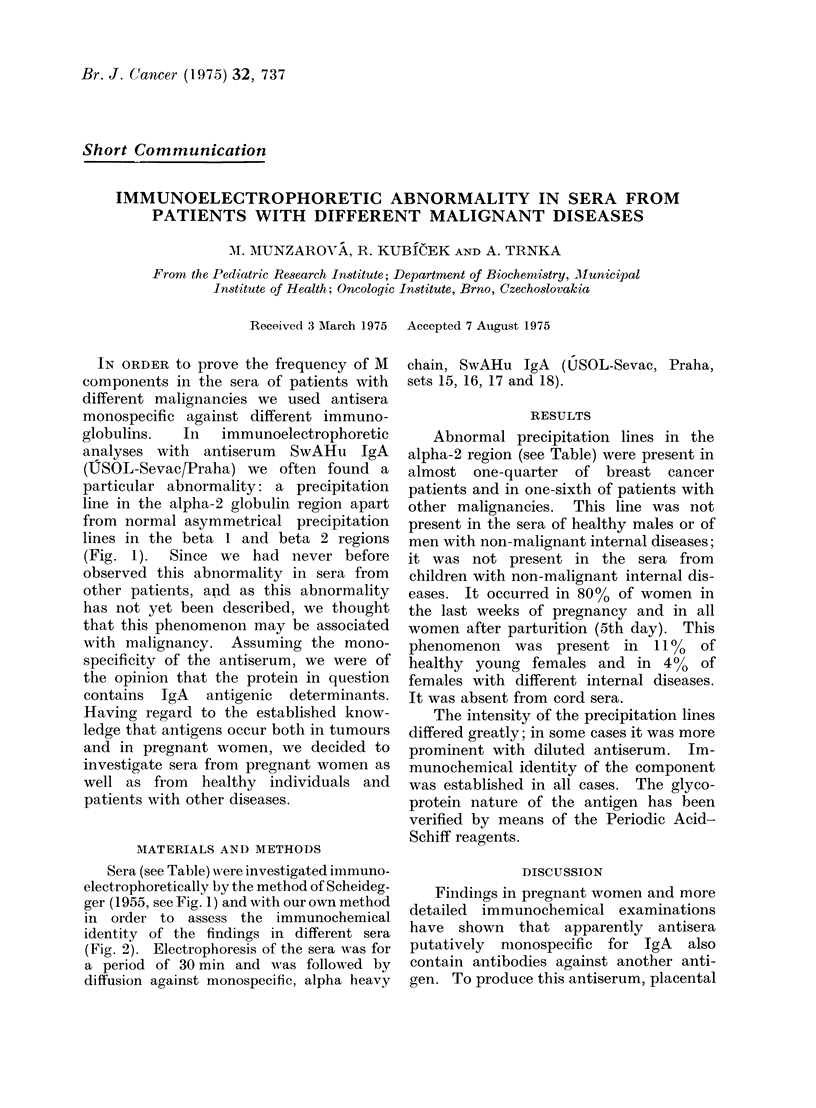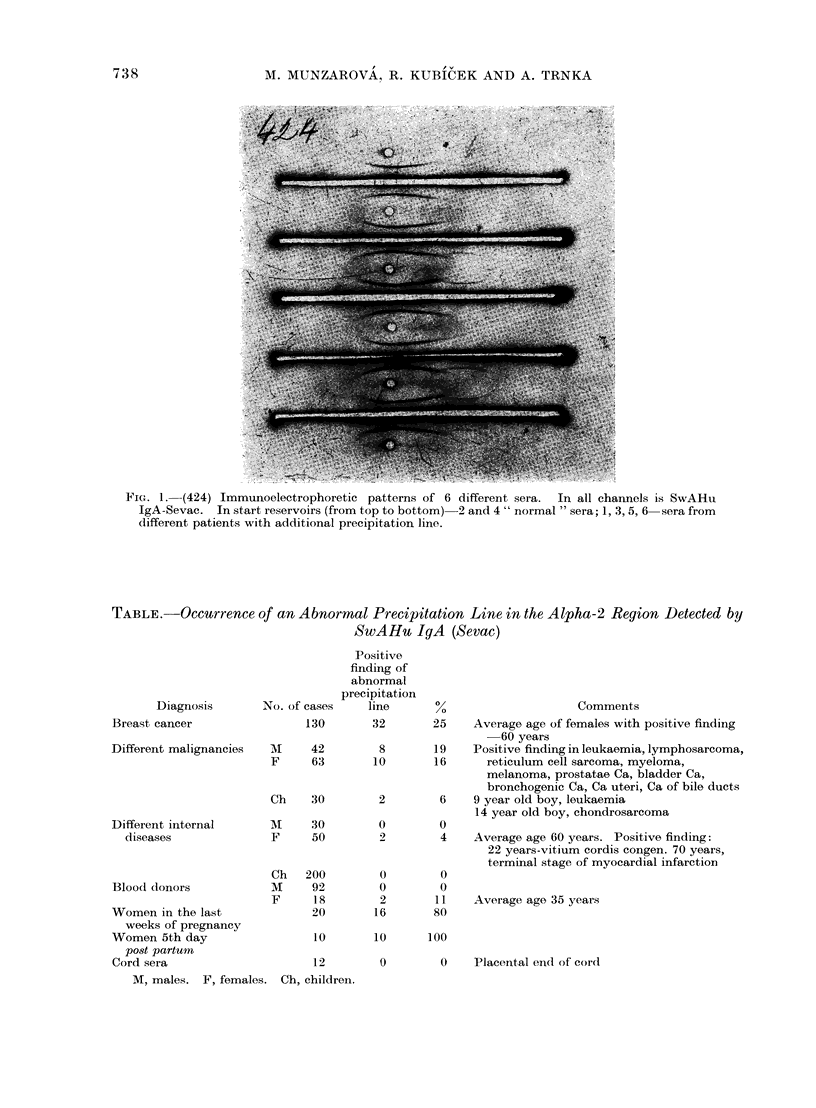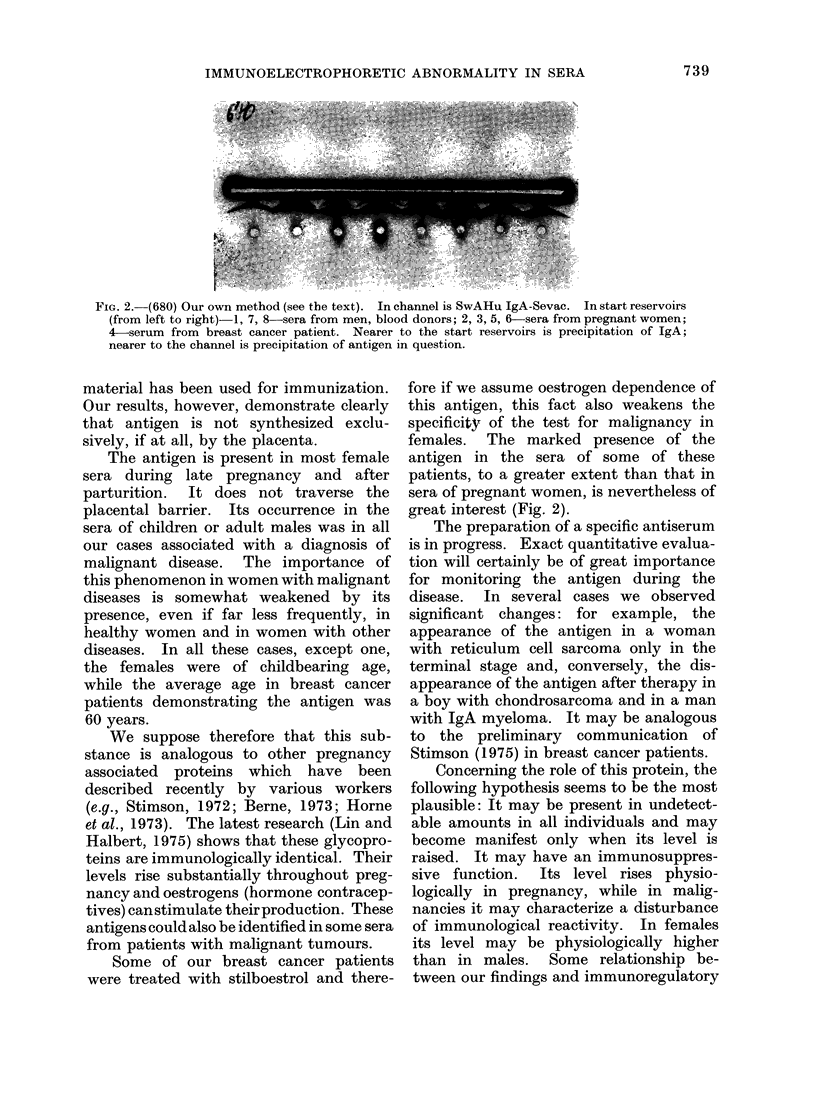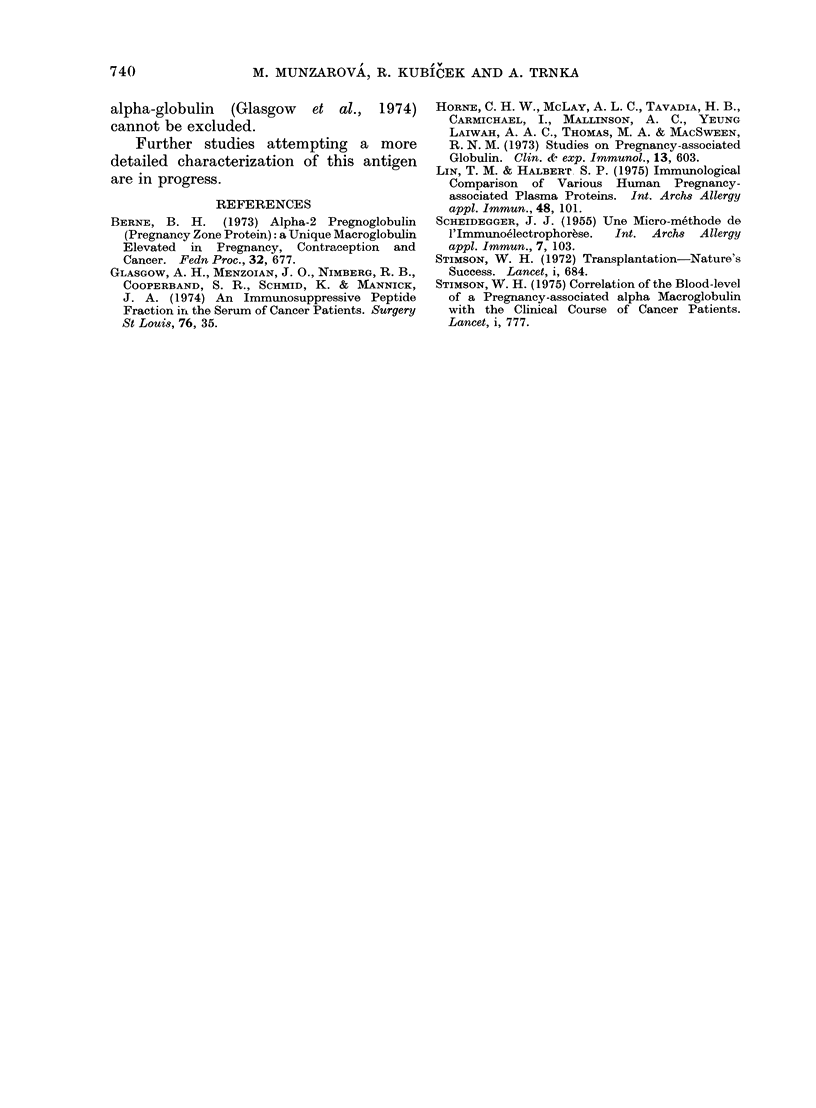# Immunoelectrophoretic abnormality in Sera from patients with different malignant diseases.

**DOI:** 10.1038/bjc.1975.285

**Published:** 1975-12

**Authors:** M. Munzarová, R. Kubícek, A. Trnka

## Abstract

**Images:**


*Br. J. Cancer* (1975) **32**, 737

***Short Communication***

### IMMUNOELECTROPHORETIC ABNORMALITY IN SERA FROM PATIENTS WITH DIFFERENT MALIGNANT DISEASES

M. MUNZAROVÁ, R. KUBÍČEK AND A. TRNKA

*From the Pediatric Research Institute; Department of Biochemistry, Municipal Institute of Health; Oncologic Institute, Brno, Czechoslovakia*

Received 3 March 1975 Accepted 7 August 1975

IN ORDER to prove the frequency of M components in the sera of patients with different malignancies we used antisera monospecific against different immunoglobulins. In immunoelectrophoretic analyses with antiserum SwAHu IgA (ÚSOL-Sevac/Praha) we often found a particular abnormality: a precipitation line in the alpha-2 globulin region apart from normal asymmetrical precipitation lines in the beta 1 and beta 2 regions (Fig. 1). Since we had never before observed this abnormality in sera from other patients, and as this abnormality has not yet been described, we thought that this phenomenon may be associated with malignancy. Assuming the monospecificity of the antiserum, we were of the opinion that the protein in question contains IgA antigenic determinants. Having regard to the established knowledge that antigens occur both in tumours and in pregnant women, we decided to investigate sera from pregnant women as well as from healthy individuals and patients with other diseases.

### MATERIALS AND METHODS

Sera (see Table) were investigated immunoelectrophoretically by the method of Scheidegger (1955, see Fig. 1) and with our own method in order to assess the immunochemical identity of the findings in different sera (Fig. 2). Electrophoresis of the sera was for a period of 30 min and was followed by diffusion against monospecific, alpha heavy chain, SwAHu IgA (ÚSOL-Sevac, Praha, sets 15, 16, 17 and 18).

### RESULTS

Abnormal precipitation lines in the alpha-2 region (see Table) were present in almost one-quarter of breast cancer patients and in one-sixth of patients with other malignancies. This line was not present in the sera of healthy males or of men with non-malignant internal diseases; it was not present in the sera from children with non-malignant internal diseases. It occurred in 80% of women in the last weeks of pregnancy and in all women after parturition (5th day). This phenomenon was present in 11% of healthy young females and in 4% of females with different internal diseases. It was absent from cord sera.

The intensity of the precipitation lines differed greatly; in some cases it was more prominent with diluted antiserum. Immunochemical identity of the component was established in all cases. The glycoprotein nature of the antigen has been verified by means of the Periodic Acid–Schiff reagents.

### DISCUSSION

Findings in pregnant women and more detailed immunochemical examinations have shown that apparently antisera putatively monospecific for IgA also contain antibodies against another antigen. To produce this antiserum, placental

FIG. 1.—(424) Immunoelectrophoretic patterns of 6 different sera. In all channels is SwAHu IgA-Sevac. In start reservoirs (from top to bottom)—2 and 4 " normal " sera; 1, 3, 5, 6—sera from different patients with additional precipitation line.

TABLE.—*Occurrence of an Abnormal Precipitation Line in the Alpha-2 Region Detected by SwAHu IgA (Sevac)*

| Diagnosis | | No. of cases | Positive finding of abnormal precipitation line | % | Comments |
|---|---|---|---|---|---|
| Breast cancer | | 130 | 32 | 25 | Average age of females with positive finding —60 years |
| Different malignancies | M | 42 | 8 | 19 | Positive finding in leukaemia, lymphosarcoma, reticulum cell sarcoma, myeloma, melanoma, prostatae Ca, bladder Ca, bronchogenic Ca, Ca uteri, Ca of bile ducts |
| | F | 63 | 10 | 16 | |
| | Ch | 30 | 2 | 6 | 9 year old boy, leukaemia<br>14 year old boy, chondrosarcoma |
| Different internal diseases | M | 30 | 0 | 0 | |
| | F | 50 | 2 | 4 | Average age 60 years. Positive finding: 22 years-vitium cordis congen. 70 years, terminal stage of myocardial infarction |
| | Ch | 200 | 0 | 0 | |
| Blood donors | M | 92 | 0 | 0 | |
| | F | 18 | 2 | 11 | Average age 35 years |
| Women in the last weeks of pregnancy | | 20 | 16 | 80 | |
| Women 5th day *post partum* | | 10 | 10 | 100 | |
| Cord sera | | 12 | 0 | 0 | Placental end of cord |

M, males. F, females. Ch, children.

FIG. 2.—(680) Our own method (see the text). In channel is SwAHu IgA-Sevac. In start reservoirs (from left to right)—1, 7, 8—sera from men, blood donors; 2, 3, 5, 6—sera from pregnant women; 4—serum from breast cancer patient. Nearer to the start reservoirs is precipitation of IgA; nearer to the channel is precipitation of antigen in question.

material has been used for immunization. Our results, however, demonstrate clearly that antigen is not synthesized exclusively, if at all, by the placenta.

The antigen is present in most female sera during late pregnancy and after parturition. It does not traverse the placental barrier. Its occurrence in the sera of children or adult males was in all our cases associated with a diagnosis of malignant disease. The importance of this phenomenon in women with malignant diseases is somewhat weakened by its presence, even if far less frequently, in healthy women and in women with other diseases. In all these cases, except one, the females were of childbearing age, while the average age in breast cancer patients demonstrating the antigen was 60 years.

We suppose therefore that this substance is analogous to other pregnancy associated proteins which have been described recently by various workers (*e.g.*, Stimson, 1972; Berne, 1973; Horne *et al.*, 1973). The latest research (Lin and Halbert, 1975) shows that these glycoproteins are immunologically identical. Their levels rise substantially throughout pregnancy and oestrogens (hormone contraceptives) can stimulate their production. These antigens could also be identified in some sera from patients with malignant tumours.

Some of our breast cancer patients were treated with stilboestrol and therefore if we assume oestrogen dependence of this antigen, this fact also weakens the specificity of the test for malignancy in females. The marked presence of the antigen in the sera of some of these patients, to a greater extent than that in sera of pregnant women, is nevertheless of great interest (Fig. 2).

The preparation of a specific antiserum is in progress. Exact quantitative evaluation will certainly be of great importance for monitoring the antigen during the disease. In several cases we observed significant changes: for example, the appearance of the antigen in a woman with reticulum cell sarcoma only in the terminal stage and, conversely, the disappearance of the antigen after therapy in a boy with chondrosarcoma and in a man with IgA myeloma. It may be analogous to the preliminary communication of Stimson (1975) in breast cancer patients.

Concerning the role of this protein, the following hypothesis seems to be the most plausible: It may be present in undetectable amounts in all individuals and may become manifest only when its level is raised. It may have an immunosuppressive function. Its level rises physiologically in pregnancy, while in malignancies it may characterize a disturbance of immunological reactivity. In females its level may be physiologically higher than in males. Some relationship between our findings and immunoregulatory alpha-globulin (Glasgow *et al.*, 1974) cannot be excluded.

Further studies attempting a more detailed characterization of this antigen are in progress.

### REFERENCES

Berne, B. H. (1973) Alpha-2 Pregnoglobulin (Pregnancy Zone Protein): a Unique Macroglobulin Elevated in Pregnancy, Contraception and Cancer. *Fedn Proc.*, **32**, 677.

Glasgow, A. H., Menzoian, J. O., Nimberg, R. B., Cooperband, S. R., Schmid, K. & Mannick, J. A. (1974) An Immunosuppressive Peptide Fraction in the Serum of Cancer Patients. *Surgery St Louis*, **76**, 35.

Horne, C. H. W., McLay, A. L. C., Tavadia, H. B., Carmichael, I., Mallinson, A. C., Yeung Laiwah, A. A. C., Thomas, M. A. & MacSween, R. N. M. (1973) Studies on Pregnancy-associated Globulin. *Clin. & exp. Immunol.*, **13**, 603.

Lin, T. M. & Halbert, S. P. (1975) Immunological Comparison of Various Human Pregnancy-associated Plasma Proteins. *Int. Archs Allergy appl. Immun.*, **48**, 101.

Scheidegger, J. J. (1955) Une Micro-méthode de l'Immunoélectrophorèse. *Int. Archs Allergy appl. Immun.*, **7**, 103.

Stimson, W. H. (1972) Transplantation—Nature's Success. *Lancet*, i, 684.

Stimson, W. H. (1975) Correlation of the Blood-level of a Pregnancy-associated alpha Macroglobulin with the Clinical Course of Cancer Patients. *Lancet*, i, 777.